# Effects of Antibiotic Consumption on Children 2-8 Years of Age Developing Asthma

**DOI:** 10.4178/epih/e2014006

**Published:** 2014-07-04

**Authors:** Hamid Reza Khalkhali, Sima Oshnouei, Shaker Salarilak, Mohammadhossein Rahimi Rad, Mohammad Karamyar, Javad Khashabi

**Affiliations:** 1 Inpatient Safety Research Center, Faculty of Medicine, Urmia University of Medical Sciences, Urmia, Iran; 2 Department of Biostatistics and Epidemiology, Faculty of Medicine, Urmia University of Medical Sciences, Urmia, Iran; 3 Reproductive Health Research Center, Urmia University of Medical Sciences, Urmia, Iran; 4 Department of Public Health, Faculty of Medicine, Tabriz Branch, Islamic Azad University, Tabriz, Iran; 5 Department of Respiratory Medicine, Faculty of Medicine, Urmia University of Medical Sciences, Urmia, Iran; 6 Department of Pediatrics, Motahari Hospital, Faculty of Medicine, Urmia University of Medical Sciences, Urmia, Iran; 7 Department of Pediatrics, Faculty of Medicine, Urmia University of Medical Sciences, Urmia, Iran

**Keywords:** Antibiotic use, Childhood asthma, Case-control studies

## Abstract

**OBJECTIVES:**

Antibiotic exposure in children is a possible contributor to the increasing asthma prevalence in several countries. The present study aimed to investigate the association between antibiotic exposure and the risk of developing childhood asthma at 2-8 years of age.

**METHODS:**

A case-control study was undertaken among children aged 2-8 years old between March and September 2010 in the Urmia district in the northwest of Iran. The cases were doctor-diagnosed asthmatic children based on Global Initiative for Asthma criteria (n=207), and the controls were children without respiratory symptoms (n=400) selected by frequency matching by age and gender. Clinical data including antibiotic exposure was collected by a validated and reliable questionnaire, which was completed by interviewing parents/guardians.

**RESULTS:**

Antibiotic consumption during the first year of life increased the odds ratio [OR] of asthma symptoms at 2-8 years of age (crude OR, 2.26; 95% confidence interval [CI], 1.53-3.35; p<0.01), and the strength of association was similar after adjusting for a family history of asthma or atopic disorder, preterm delivery, birth order, and delivery method (adjusted OR, 1.91; 95% CI, 1.27-2.88; p=0.03).

**CONCLUSIONS:**

Our study suggests that antibiotic consumption in children was associated with an increased risk of childhood asthma, and an additional confirmative study is needed.

## INTRODUCTION

Asthma is the most common chronic disease in childhood, affecting up to 300 million people in the world [1]. During the last decades, many lifestyle behaviors including, dietary patterns, and health behaviors related with life style have changed the pattern of asthma and other respiratory disorders across the world, particularly in developing countries [2].

Antibiotics are broadly used to treat various infections in childhood. A great deal of evidence suggests that gastrointestinal flora plays a prominent role in developing a healthy immune system and resistance to allergic sensitization [3]. Newer studies have shown that antibiotic consumption in early life may increase the development of asthma by reducing the protective effect of bacterial infections and disturbance of the normal gut bacterial flora [4,5]. but no significant association between antibiotic exposure and developing asthma and other allergic disorders in childhood has been identified [6]. Nevertheless, decreasing excess antibiotic consumption in childhood is recommended [7].

This article reports on data drawn from a observational study investigating potential risk factors for the development of childhood asthma in subjects 2-8 years of age in the city of Urmia, in the northwest of Iran, and the primary objective was to investigate the association between antibiotic exposure and the risk of developing childhood asthma.

## MATERIALS AND METHODS

This investigation was a case-control study conducted between March and September 2010 in Urmia, Iran.

The study was approved by the Human Research Ethics Committee of Urmia University of Medical Sciences, and the questionnaire was administered by the author after verbal consent was obtained from all of the participating children’s parents.

The cases (n=207) were selected from among children 2-8 years of age who were referred to the Motahhari Children’s Polyclinic and the Asthma and Allergy Pediatric Center. To recruit incident asthmatic patients, we adopted the Global Initiative for Asthma (GINA) criteria prospectively between March and September 2010. The patient’s history of lower respiratory infections was assessed in the questionnaire in order to reduce the likelihood of reverse causation and reduce the likelihood of needing a differential diagnosis between asthma and respiratory tract infections, all of the patients with a history of severe respiratory infections during the first two years of life were excluded from the study population.

Controls (n=400) were selected from among children of the same age range whose parents did not report any history of asthma or allergic disorders. To reduce selection bias, we selected controls from hospitalized and healthy children who visited the Motahhari Children’s Polyclinic, matched by age and gender using the frequency matching method: The hospitalized controls were selected from among the patients without any allergic or respiratory disease, and the healthy controls were selected from among the children that were referred to the health centers for their routine growth monitoring. Additional controls were recruited from areas of the city in which the cases were living to reduce socioeconomic differences and other unknown confounding factors between cases and controls.

A validated questionnaire prepared by the International Study of Asthma and Allergies in Childhood (ISSAC, Phase III) was used for data collection. It was translated into the Persian language first, and data related to the history of antibiotic consumption in the first year of life and during pregnancy were collected by interviewing the parents/guardians.

All of the questionnaires were completed by one interviewer in order to enhance the reliability of the results.

Because all of the risk factors in the childhood asthma-based ISSAC hypotheses were evaluated in this study, we used procedures to identify factors related to childhood asthma.

The Pearson chi-squared test was applied to assess the association between antibiotic exposure and risk of childhood asthma. We fitted multiple logistic regression models using a stepwise selection strategy with statistically significant variables that were reported as confounding factors in previous studies including maternal smoking, history of asthma or atopic disease in the first- and second-degree relatives, duration of breastfeeding, number of older/younger siblings in the household, preterm delivery, and delivery method [8,9]. STATA version 10.0 (StataCorp, College Station, TX, USA) was used for data analysis.

## RESULTS

Antibiotics were used by 45 cases (23.7%), 43 healthy controls (14.9%), and 40 (20.5%) hospitalized control mothers of patients. Maternal antibiotic consumption during pregnancy had no significant effects on the risk of developing asthma in the study population; therefore, it was not entered in the regression model (crude odds ratio [OR], 1.20; 95% confidence interval [CI], 0.76-0.84; p=0.42).

Antibiotics were used by 120 cases (71.9%) and 205 controls (56.7 and 49.2% of hospitalized and healthy controls, respectively) during the first year of life, and this had a significant effect on the risk of asthma (p<0.01). All of mothers was not report history of smoking during pregnancy (Table 1).

Table 2 shows crude and adjusted odds ratios for the association of antibiotic consumption and other important risk factors during the first year of life and the risk of asthma in children 2-8 years of age. Interactions between antibiotic consumption and other asthma risk factors were not statistically significant and were thus removed in a stepwise selection method (p>0.05). Antibiotic consumption during the first year of life increased the risk of asthma symptoms at 2-8 years of age (crude OR, 2.26; 95% CI, 1.50-3.35; p<0.01 and adjusted OR, 1.91; 95% CI, 1.27-2.88; p<0.05).

## DISCUSSION

Antibiotic exposure during the first year of life significantly increased the risk of asthma at 2-8 years of age. Even though antibiotic exposure during fetal development was higher among the cases than the control groups (23.7% of cases vs. 22.6% of controls), the difference was not statistically significant (p=0.57).

Although our study design has the same limitations of other case-control studies, we tried to reduce interviewer bias by having a single trained nurse conduct all interviews. The standard GINA criteria were used to avoid the common differential in childhood asthma. Antibiotic consumption during the first year of life increased odds of asthma at 2-8 years, and this association was persistent even after adjusting for significant risk factors in the multiple regressions.

Our antibiotic hypotheses were based on Wickens’ study, which showed an associated risk of asthma, allergic rhino-conjunctivitis, and atopic eczema in patients who were administered antibiotics in early childhood [10]. ISSAC Phase III has investigated many factors that have been hypothesized to increase the risk of asthma and other allergic disorders in childhood. That study investigated the effect of antibiotic consumption and the subsequent increased risk of these allergic disorders in 193, 412 children from 71 centers in 29 countries using cross-sectional surveys. Although it was found that acetaminophen use was associated with an increased risk of asthma and other allergic disorders, the same data set yielded a much stronger association for antibiotics compared to acetaminophen exposure [11].

A recent review study by Murk and colleagues has suggested two probable confounders for such relationships: “reverse causality” and “protopathic bias” [12]. A significant association has also been reported between antibiotic consumption and increased risk of childhood asthma through a prospective study and a sensitivity analysis has also been performed to evaluate protopathic bias [13,14], Kozyrskyj et al. [15] have shown this association using a cohort study of 13,116 children (OR, 1.86; 95% CI, 1.02-3.37), in which the risk of asthma was stronger in children who had received more courses of antibiotics (OR,1.46; 95% CI, 1.14-1.88). Risnes and colleagues have concluded that antibiotic consumption increased the risk of developing childhood asthma even in children not having experienced respiratory tract infections and in children whose disease was diagnosed after 3 years of age and all cases whose disease was diagnosed before 2 years of age were excluded from the case group to reduce the risk of protopathic bias well as the risk of reverse causation [14].

In conclusion, we found the odds of childhood asthma to be associated with antibiotic use in children. We recommend a further prospective cohort studies on the association between antibiotic use in early life and childhood asthma.

## Figures and Tables

**Table 1.** Distribution of demographic and other features in the study population

Variables		Cases	Healthy controls	Hospitalized controls	p-value^1^
Antibiotic use during the first year of life	No	47 (28.1)	84 (43.3)	98 (50.8)	< 0.01
	Yes	120 (71.9)	110 (56.7)	95 (49.2)	
Exposure to antibiotics during pregnancy	No	145 (76.3)	130 (75.1)	155 (79.5)	0.57
	Yes	45 (23.7)	43 (14.9)	40 (20.5)	
Sex	Male	121 (58.1)	113 (56.5)	115 (57.5)	0.91
	Female	85 (41.3)	87 (43.5)	85 (41.3)	
Living area	Urban	195 (95.6)	179 (89.9)	181 (91.0)	0.08
	Rural	9 (4.4)	20 (10.1)	18 (9.0)	
Age at time of diagnosis (yr)	2-4	230 (38.4)			
	4-6	208 (34.7)			
	6-8	161 (26.9)			
History of passive smoking (yr)	Never	147 (71.0)	150 (75.0)	140 (70.0)	0.89
	<10	34 (16.4)	30 (15.0)	32 (16.0)	
	11-19	5 (2.4)	4 (180.0)	4 (1.9)	
	≥20	21 (10.2)	16 (8.2)	24 (12.1)	
Birth order	1-2	191 (95.5)	179 (89.5)	163 (81.5)	< 0.01
	≥3	16 (4.5)	20 (10.5)	37 (18.5)	
Birth weight (g)	<2,500	15 (7.6)	10 (5.2)	10 (5.2)	0.51
	≥2,500	182 (92.4)	183 (94.8)	183 (94.8)	
Delivery method	Vaginal	84 (40.6)	91 (45.7)	116 (58.0)	< 0.01
	Caesarean	123 (59.4)	108 (54.3)	84 (42.0)	
Preterm delivery (≤37 wk)	No	174 (84.5)	178 (89.4)	189 (94.5)	< 0.01
	Yes	32 (15.5)	21 (10.6)	11 (5.5)	
History of mother smoking	No	203 (99.5)	197 (98.5)	198 (99.0)	0.54
	Yes	1 (0.5)	3(1.5)	0 (0)	
History of asthma or atopic disorders in the first or second degree relatives	None	52 (25.5)	86 (43.2)	119 (59.5)	< 0.01
	Asthma	44 (21.6)	30 (15.1)	15 (7.5)	
	Atopic disorders	78 (38.2)	64 (32.2)	58 (29)	
	Both	30 (14.7)	19 (9.5)	8 (4)	

Values are presented as number (%). ^1^Chi squared or Fisher’s exact test.

**Table 2.** Distribution of antibiotic consumption and other risk factors during the first year of life and risk of asthma at 2-8 years

Variables	Crude OR (95% CI)	p-value^1^	Adjusted OR (95% CI)	p-value^1^
Antibiotic use during the first year of life				
No	1.00	<0.01	1.00	<0.05
Yes	2.26 (1.53 -3.35)		1.91 (1.27-2.88)	
Adjusted variables				
History of asthma or atopic disorders in the first- or second-degree relatives				
None	1.00	<0.01	1.00	<0.01
Asthma	3.85 (2.30-6.45)		3.36 (1.83-5.81)	
Atopic disorders	2.52 (1.66-3.82)		2.65 (1.67-4.20)	
Asthma/atopic disorders	4.38 (2.39-8.00)		4.43 (2.31-8.49)	
Preterm delivery (≤37 wk)				
No	1.00	<0.01	1.00	<0.01
Yes	2.10 (1.25-3.55)		2.21 (1.23-3.99)	
Birth order				
1-2	1.00	0.01	NA	
≥3	0.7 (0.52-0.94)			
Delivery method				
Vaginal	1.00	<0.01	NA	
Caesarean	1.57 (1.12-2.21)			

OR, odds ratio; CI, confidence interval; NA, not applicable. ^1^p-value in the univariate regression model.
